# Scientific Societies

**Published:** 1886-08

**Authors:** 


### SCIENTIFIC SOCIETIES.

In England and on the Continent, to belong to a recognized scientific society is a mark of distinction. The members of the Royal, the Geographical, the Microscopical, the Linnæan, and other similar organizations, append the initials to their name, and the F. R. S., F. R. M. S., F. L. S., etc., are as well known as our own M. D., or LL. D. They are a distinction because they have never been cheapened by indiscriminate bestowal. A man must have a recognized scientific standing to secure membership, and the dilettanti and the pseudo-scientists are rigidly excluded.

We might have such societies in America if they were properly conducted. In dentistry a number of organizations have been effected with a grandiloquent title attached to membership. But the trouble was that they usually were instituted by men who had no particular professional standing, or they were soon invaded by dentists with shady reputations, and their good name was tainted at the outset. The time will come when Americans will have leisure to turn their attention to other than practical every-day matters, and when the better class of professional men will band themselves into societies which shall command the respect of all the world. In the meantime, we may well rest content with our present reputation for practical horse-sense and inventive ingenuity.

